# New products

**DOI:** 10.1155/S1463924688000124

**Published:** 1988


Journal of Automatic Chemistry, Vol. 10, No. 1 (January–March 1988), pp. 62–64

### New products

### CD-ROM data-base

Quicker medical treatment is just one of the advantages being offered by the latest laser disc technology. Comprehensive, drug and other related information is available on a compact disc data-base, *Micromedex*, produced by Micromedex Inc., an American medical information company.

The *Micromedex CD-ROM* (Compact Disc–Read Only Memory) data-base has just been released in the UK and Micromedex has appointed Microinfo to market and distribute the new CD-ROM service.

The *Micromedex* data-base has five information sources:

- POISINDEX – A toxicology data-base which identifies substances, referencing them by generic chemical, street or slang terms and giving protocols for treatment.
- DRUGDEX – A drug information system giving evaluations for more than 3700 US Food and Drug Administration-approved, non-US and over-the-counter drugs.
- MARTINDALE: The extra Pharmacopoeia – Summary of 5100 properties, actions, and uses of drugs and medicines in clinical use throughout the world.
- TOMES – Information on industrial and environmental toxicology, as well as occupational medicine.
- EMERGINDEX – A clinical information system which presents medical data for clinicians in the emergency room.
- IDENTIDEX – A data-base identifying more than 20 000 tablets and capsules using the manufacturer's imprint coding and the pill's colour and physical description.

*Details from Microinfo Ltd, PO Box 3, Omega Park, Alton, Hampshire GU34 2PG, UK.*

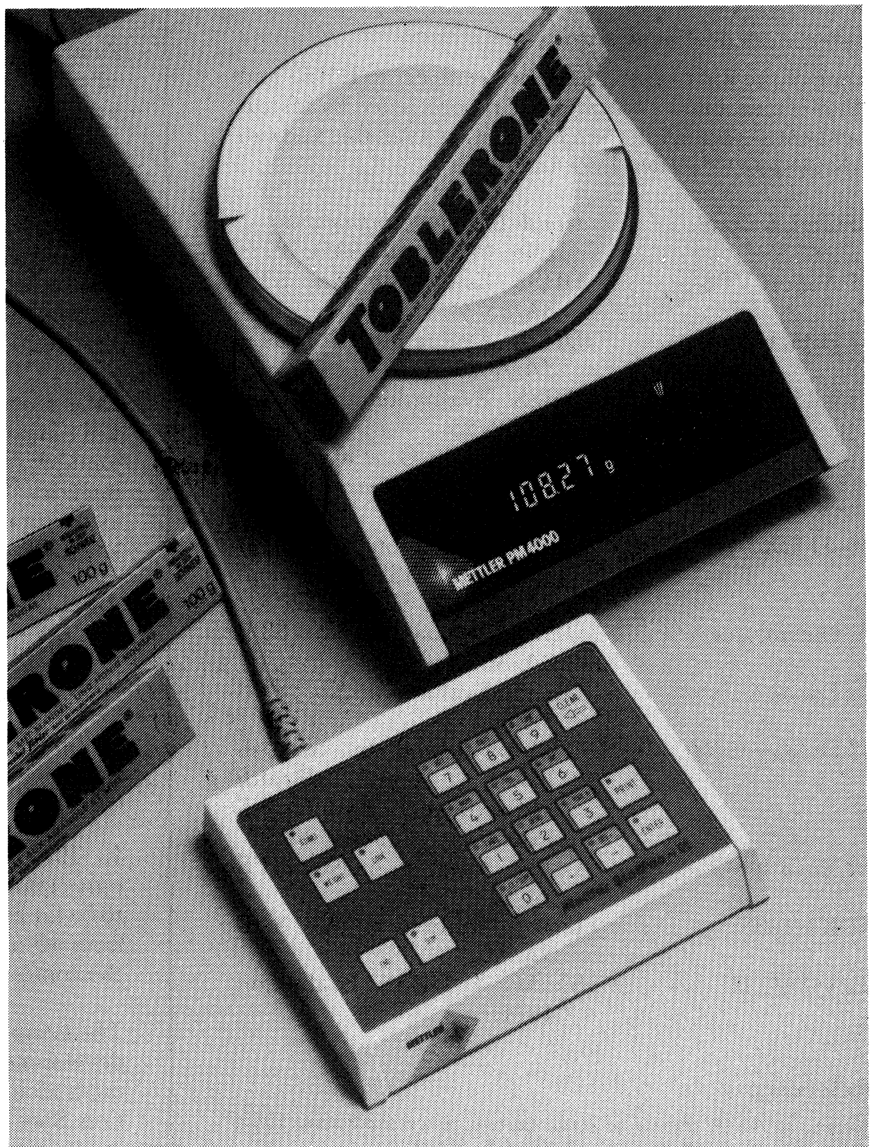

*With the new Mettler StatPac-M random samples can be checked next to the filling machine, and at the same time the weight readings can be statistically evaluated and the results printed-out. The system also continuously logs the results of the random checks, so providing records of the totals. The read-out appears on the display of the balance. (Details from Mettler Instrumente AG, CH 8606, Greifensee, Switzerland.)*

### Formulating

The Mettler LabWare Formulating is a new, user-friendly software package that improves the productivity of routine jobs involving a lot of calculating and recording with conventional methods. The package is for use with the Epson PX-4 handheld computer (called HX-40 in the USA) and either one or two Mettler balances in production, filling plants, stores and laboratories. It is remarkably easy to use. All the data needed for the mixture are keyed in and stored at the touch of a button. No knowledge of programming or data processing is required.

The computer's display guides the user through the stored formulas. When the formula has been selected, its code is keyed in and the nominal weight of the mix to be prepared is specified. As the items are weighed in, the weight of each ingredient shown on the display of the balance decreases towards zero.

The user can define a weighing-in tolerance for each ingredient of a formula. If this is exceeded while dispensing, the system announces the fact both acoustically and visually. The system has other interesting features such as tare preset (manual or automatic), a totals register and the ability to protect the stored formulae with a password.

The RAM will hold up to 200 ingredients and 10 formulae each of 20 ingredients. With a RAM disk, tape cassette or floppy disk it is possible to store up to 100 formulae.

With a printer connected, this compact system produces detailed records of all dispensing operations. As well as the final report, it is possible to print out the stored standard formulae and also catalogues of formulae and ingredients.

*Details from Mettler Instrumente AG, CH 8606 Greifensee, Switzerland.*

### Density and refractive measurements

Working in association with Allied Data, of Houghton-Le-Spring, Tyne and Wear, PS Analytical has configured and installed an Automated Refractive Index Measurement System. Samples of varying viscosity and nature are held in sealed vials on a PSA 20·080 Autosampler. This is linked to the control computer using the PSA RS232 standard interface. This computer board is also modified to control an additional piston pump to withdraw the sample and a wash pump to deliver the wash medium to the wash-out. The control computer sends commands to the system to select sample and withdraw it completely through the two analytical devices, so that stable and reproducible readings are obtained. The computer sends a command to the autosampler computer system, it is executed and the status of the Autosampler returned to the control computer.

The computer then communicates to both an Anton Paar DMA-45 with RS232 interface and to an Index RIC 1 RI Detector also along an RS232 interface. Both units use different protocols, but these are handled by a hardware/software configuration developed by Allied Data Ltd. A completely automated system with control and selection of sample position and collection of data is provided. By linking the computer to a host computer with reference data on the products under test, it is also possible to check sample specification on-line and use known test criteria, provided by the host computer to set up the Density/RI measurement collection cycle.

*Further details from PS Analytical, Arthur House, Far North Building, Cray Avenue, Orpington, Kent BR5 3TR, UK. Tel.: 0689 31632.*

### Storing titration methods with a PC

The VIA 101 (Radiometer) package enables titration parameters to be transferred between the VIT90 Video Titrator and a PC. Individual titration methods can be associated with application information. As an example, the delivered diskette contains titration parameters and application information covering 10 different pharmaceutical analyses.

The product is delivered complete with the necessary cable for any IBM PC/XT/AT compatible computer. The description is delivered both in written form and as part of an up-start program. Use of the program is straightforward and does not require any computer knowledge.

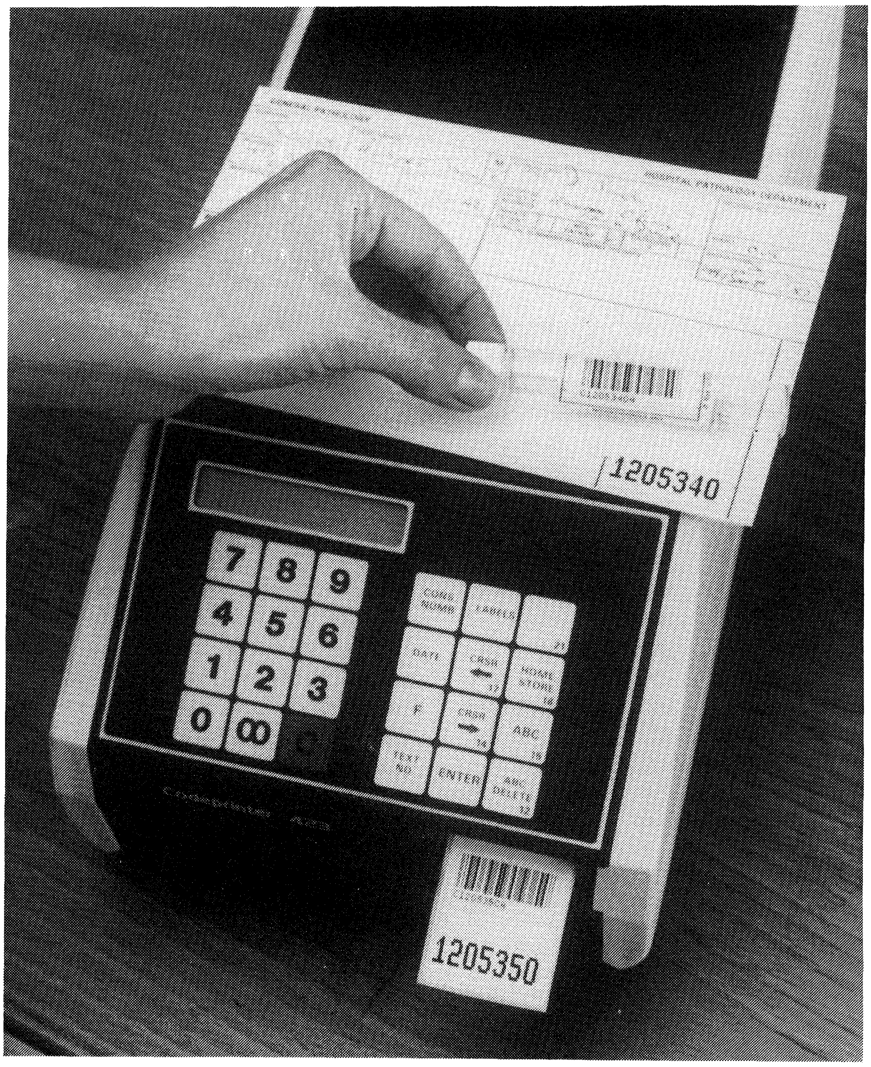

*At its simplest the Kelgray Codeprinter produces two-part labels – the first part including a bar code for tagging the specimen and the other incorporating a corresponding digit number for the patient's record card.* *(See p. 64.)*

The product is particularly useful for backing up titration methods, or for making a temporary exchange of some of the VIT90 titration methods.

Additional texts can be written using the operator's preferred text editor.

A method index is automatically generated.

*Further information on the VIA101 or the TitraLab titration system may be obtained from Radiometer Analytical A/S, Krogshøjvej 49, DK-2880 Bagsværd, Denmark.*

### Mercury system

A fully automated fluorescence system for the analysis of mercury at extremely low levels has recently been introduced by PS Analytical. Working with Spex Industries UK, PS Analytical has modified a fluorescence spectrometer to provide optimum signal output from mercury vapour produced by the hydride generator. A PS Analytical applications note describes the analytical conditions required and illustrates the level of results which can be achieved, both with standards and with real samples.

Automation is further completed by linking through a hardware/software system to an IBM compatible machine. The computer not only controls the instruments incorporated into the system, but also calculates the results by reference to a standard calibration graph.

The software allows the calculations to be checked using standard graph packages and to integrate the results into standard IBM compatible software packages, i.e. spreadsheets, word processing for reports, transmission and presentation.

*Further details from PS Analytical (above).*

### Lab 'Identikit' follows Swedish success

A low-cost 'bar code' printing system for identifying specimens used in hospital laboratory blood, urine, and tissue analysis is being introduced to Britain by Kelgray Products Ltd (Kelgray) of Crawley, Sussex following successful application in one of Sweden's leading hospitals.

Kelgray's 'Codeprinter 423' is an intelligent and co-operative desk-top printer providing for instant and silent thermal printing, as and when required, of labels or tags with operator-variable text and layout. High definition four dots per mm quality – important for accurate bar code scanning – on a two-part self-adhesive label, produces a bar-code sticker for the specimen container and a conventional digit label for the patient's reference card or other documentation.

The operator can also vary the text using different fonts and employ horizontal or vertical printing, if desired, within his chosen layout.

By means of an LCD readout, various coding keys and a numeric keypad, the Codeprinter can be user-programmed for various formats, designs and texts. A memory stores many facilities for sequential numbering and datacoding. Therefore a single Codeprinter can print weekly or monthly stocks of a variety of sequentially numbered and bar-coded labels and tags.

*Further information from Bill Smith, Kelgray Products Ltd, Kelgray House, Spindle Way, Crawley, West Sussex RH10 1TH, UK. Tel.: 0293 518733.*

### Class 100 clean room oven

Dage (GB) Ltd has announced availability of the RCOS-2220, a clean room oven with high particle-infiltration protection. The stainless-steel interior chamber has a completely welded liner with coved corners. It provides particle protection to Class 100 level. Temperature uniformity is maintained using a horizontal air circulation system. A solid state controller provides digital temperature indication in °C. Highly efficient heat-resistant filters are used in the filtration system which further minimizes particle penetration. The cold rolled steel outer casing is coated with an easy to clean melamine resin.

In the semiconductor development and manufacturing industry, and in medicine, the oven provides a drying or curing facility with a highly efficient particle filtration system.

Four standard sizes are available, each with two temperature ranges. The four oven capacities are 3·2, 7·6, 16·2, and 30·5 cubic feet. Temperature ranges are +70° to +200 °C, and +70° to +350 °C.

*For further details contact Eric Bailey, Dage (GB) Ltd, Intersem Division, Rabans Lane, Aylesbury, Bucks HP19 3RG. Tel.: 0296 393200.*

### Miniature temperature controller

A miniature, digital indicating, microprocessor-based temperature controller is available in Europe from the CAL 9000 components. With fully adjustable, three term PID (proportional, integral and derivative) control and ready-to-run push-button sensor selection, the instrument can operate from any standard thermocouple or PT100/RTD sensor.

The CAL 9000 sells at well below the cost of larger DIN format units and can be easily adapted to either 96 × 96 mm or 96 × 48 mm panel cut-outs, where required. The specification includes user ranging, an electronic scale stop and two level tamperproof security. In addition, a second slave output gives the option of alarm signals in high, low or out of limits mode or can be selected as a proportional cool channel. Also, an adjustable, permanent error display can be selected in normal, low or high resolution and the instrument is keyable in either °C or °F.

*For further information contact: Brian Hall, Controls & Automation Ltd, Bury Mead Road, Hitchin, Hertfordshire SG5 1RT, UK. Tel.: 0462 36161.*

### PS Analytical appointed distributor for Cetac range in Europe

Cetac Industries products are now available from the PS Analytical agency network throughout Europe. This appointment as European distributor is by arrangement with the sole worldwide distributors, Questron Inc. of Princeton, New Jersey, USA.

*Further information from PS Analytical (as above).*

